# Influence of fiber type and carbohydrase supplementation on nutrient digestibility, energy and nitrogen balance, and physiology of sows at mid and late gestation

**DOI:** 10.1093/jas/skad390

**Published:** 2023-11-28

**Authors:** Thomas A Crome, Mark A Giesemann, Hannah E Miller, Amy L Petry

**Affiliations:** Department of Animal and Food Sciences, Texas Tech University, Lubbock, TX 79409, USA; Adisseo USA, Inc, Alpharetta, GA 30022, USA; Division of Animal Sciences, University of Missouri, Columbia, MO 65201, USA; Division of Animal Sciences, University of Missouri, Columbia, MO 65201, USA

**Keywords:** Carbohydrases, digestibility, gestating sows, inflammation, insoluble fiber, soluble fiber

## Abstract

Carbohydrase supplementation in grow-finish pig diets improves energy, nutrient digestibility, and gastrointestinal function, but their efficacy in gestation diets is understudied. The experimental objective was to evaluate the efficacy of a multicarbohydrase to improve digestion, energetics, and various physiological functions in gestating sows fed soluble and insoluble fiber diets. On day 28 of gestation, 36 sows (186 ± 4.6 kg body weight), blocked by parity, were randomly assigned to a 2 × 2 factorial arrangement of dietary treatments (*n* = 9). Factors included fiber type of insoluble (**IF**; 20% dried distiller grains with solubles) or soluble fiber (**SF**; 20% sugar beet pulp) and with (**+**) or without (−) enzyme (0.05%, Rovabio Advance P10; Adisseo, Antony, France). Diets were fed from days 28 to 109 of gestation at a feeding level of 2.1 kg (SID-Lys 11 g/d and 4.5 net energy-Mcal/d). Two separate 9-d metabolism periods were conducted on days 50 to 59 (**mid**) and 99 to 108 (**late**) of gestation. During each period, days 1 to 3 served as an adaptation period, days 4 to 7 total urine and feces were collected (96-h) and followed by a 48-h lactulose-mannitol study. Serum and plasma were collected on days 50 and 99. Data were analyzed as repeated records using a linear mixed model with block as a random effect and fiber type, enzyme, and period and their interactions as fixed effects. Sows fed SF+ had increased serum IL-1ra (Fiber × Enzyme *P* = 0.035), and IL-2 (Fiber × Enzyme *P* = 0.042). In the presence of IF, multicarbohydrases increased serum lipopolysaccharide-binding protein, but not when supplemented with SF (Fiber × Enzyme *P* = 0.028). Circulating IL-8 and TNF-α were decreased in sows fed multicarbohydrases (*P < *0.05). Multicarbohydrase supplementation increased the apparent total tract digestibility (**ATTD**) of gross energy (**GE**), dry matter, and neutral detergent fiber by 2.8%, 3.4%, and 8.3%, respectively (*P* < 0.05). Compared to IF−, the ATTD of hemicellulose was 5.3% greater in sows fed IF+ but did not differ from SF− and SF+ (Fiber × Enzyme *P* = 0.037). Sows fed IF+ had the greatest ATTD of insoluble dietary fiber (Fiber × Enzyme *P* = 0.011). Sows fed multicarbohydrases excreted less energy in their urine (519 vs. 469 GE kcal/d; Enzyme *P = *0.033) and in their feces (985 vs. 900 GE kcal/d; Enzyme *P = *0.003). This resulted in an improvement in both digestible energy (Enzyme *P* < 0.01) and metabolizable energy (Enzyme *P* = 0.041), irrespective of fiber type. In conclusion, multicarbohydrase supplementation increased the digestibility and energetic contribution of fiber, irrespective of adaptation time or fiber type, but modulation of inflammatory responses was unique to dietary fiber type.

## Teaser

This study investigates the impact of multicarbohydrase supplementation in gestating sow diets, highlighting its potential to enhance nutrient digestibility, increase metabolizable energy, and reduce inflammatory responses. The research highlights the importance of considering dietary fiber type when using multicarbohydrases, emphasizing their role in improving gastrointestinal function and altering inflammatory responses in gestating sows.

## Introduction

In the U.S., ~15% to 20% of the cost to raise a pig to market is derived from feed costs associated with the sow herd. Fibrous feedstuffs are commonly used in gestating sow diets because of their bulk density and energy dilution effect, which aids in satiety and mitigating behavioral vices associated with feed restriction (Agyekum and Nyachoti, 2017; Huang et al., 2020). Swine diets rich in dietary fiber (**DF**) also beneficially modulate gastrointestinal microbiota, increase insulin sensitivity, and improve systemic health (Jha et al., 2019; Melo-Duràn et al., 2019). However, most of the cost-effective DF sources in the U.S. are often insoluble corn co-products that are poorly fermentable, increase rate of passage, dilute dietary energy, and reduce nutrient digestibility (Acosta et al., 2020; Petry and Patience, 2020). Strategies that improve the energetic contribution and capture the beneficial effects of DF from corn co-products are warranted to decrease feed costs and improve sow health.

Supplementing gestation diets with substrate-specific carbohydrase enzymes could be a potential solution to improve the energetic contribution of DF. Carbohydrases have the potential to offset the antinutritive effects associated with corn-based DF while providing health benefits such as altering immune function (Chen et al., 2020), improving gut barrier integrity (Tiwari et al., 2018), and upregulating bifidogenic bacterial communities (Petry and Patience, 2020; Petry et al., 2020). They may also bridge the gap between the economics of including corn co-products in diets and the health benefits of rapidly fermentable DF (Bedford, 2018). However, there is minimal knowledge of carbohydrase efficacy and mechanism in sow diets. Therefore, the experimental objective was to evaluate if the effectiveness and mechanisms associated with multicarbohydrase supplementation in the grow-finish pig are translatable to gestating sows. It was hypothesized that irrespective of fiber type, multicarbohydrase supplementation will improve metabolizable energy (ME), nutrient digestibility, and markers of gastrointestinal function and immunity in gestating sows fed soluble and insoluble DF.

## Materials and Methods

All experimental procedures were reviewed and approved by the Texas Tech University Institutional Animal Care and Use Committee (T-21065) and adhered to guidelines for the ethical and humane use of animals for research as described by the Guide for the Care and Use of Agricultural Animals in Research and Teaching (FASS, 2010).

### Animals, housing, and experimental design

Thirty-six confirmed bred nulliparous and multiparous sows (parity 3 ± 0. 73, Camborough; PIC Inc., Hendersonville, TN) with an initial body weight of 186 ± 4.6 kg were used in three replicates of an 80-d trial. Sows were randomly assigned to 1 of 4 dietary treatments in a 2 × 2 factorial arrangement on day 28 of gestation. Factors included fiber type of insoluble (**IF**; 15.1 IDF%) or soluble fiber (**SF**; 4.6 SDF%) and with (**+**) or without (**−**) multicarbohydrase supplementation (0.05%, Rovabio Advance P10; Adisseo, Antony, France). Treatment assignment was balanced among replicates and parities. Sows were housed individually in gestation stalls (1.41 m^2^) equipped with a nipple waterer, individually slotted feed trough, and concrete slatted floor. On days 50 (mid gestation) and 99 of gestation (late gestation), sows were moved to individual raised modified gestation stalls (1.41 m^2^) to permit the separate collection of feces and urine for a 9-d metabolism study. During each metabolism period, days 1 to 3 served as an environmental adaptation period, from days 4 to 7 total urine and feces were collected (96-h) and were followed by a 48-h lactulose-mannitol study.

### Diets and feeding

All diets exceeded nutritional requirements set forth by the National Research Council (2012) gestation model assuming 50 kg of maternal body weight gain and a litter size of 16 and were congruent with PIC nutrition guidelines for maternal lines. Diets were formulated with the intention of fixing the amount of insoluble or SF inclusion while letting energy float. The IF diet contained 20% inclusion of dried distiller grains with solubles (**DDGS**), whereas SF contained 20% inclusion of sugar beet pulp (**SBP**; Table 1). Soybean meal (**SBM**) was held at a 5% inclusion among diets to keep the contribution of fiber from SBM congruent among treatments. Corn gluten meal was used in SF treatments as an amino acid source with a similar composition to DDGS but reduced additional contribution of IF. For enzyme treatments, 0.05% of a multicarbohydrase product (Rovabio Advance P10; Adisseo, Antony, France) was supplemented. The multicarbohydrase was composed of 19 exogenous enzymes produced from *Talaromyces versatilis* designed to target arabinoxylan, xyloglucan, β-glucan, cellulose, pectin, and β-mannan polysaccharides as described by Cozannet et al. (2019). Both DDGS and SBP contained polysaccharides targeted by multiple enzymes within the multicarbohydrase product. Diets were manufactured as a mash, and ten representative samples of each batch were collected during manufacturing, homogenized, and stored at −20 °C for subsequent analysis.

**Table 1.** Ingredient and calculated nutrient composition of experimental diets (as-fed basis).

	Treatment^1^			
Item	IF−	IF+	SF−	SF+
Ingredient composition, %				
Corn	71.18	71.13	63.47	63.42
Corn DDGS	20.00	20.00	0.00	0.00
Sugar beet pulp	0.00	0.00	20.00	20.00
Corn gluten meal	0.00	0.00	7.83	7.83
Soybean meal	5.10	5.10	5.10	5.10
Limestone	1.72	1.72	1.22	1.22
Monocalcium phosphate 21%	0.64	0.64	0.99	0.99
Sodium chloride	0.50	0.50	0.50	0.50
Vitamin premix^2^	0.30	0.30	0.30	0.30
L-lysine HCl	0.20	0.20	0.20	0.20
Trace mineral premix^3^	0.15	0.15	0.15	0.15
Choline chloride 60%	0.15	0.15	0.15	0.15
Multicarbohydrase^4^	0.00	0.05	0.00	0.05
L-threonine	0.04	0.04	0.07	0.07
Phytase^5^	0.01	0.01	0.01	0.01
L-tryptophan	0.01	0.01	0.01	0.01
Calculated nutrient composition				
SID^6^ Lysine, %	0.52	0.52	0.52	0.52
SID TSAA^7^: Lysine	0.70	0.70	0.70	0.70
SID Threonine: Lysine	0.76	0.76	0.76	0.76
SID Tryptophan: Lysine	0.19	0.19	0.23	0.23
Ca, %	0.83	0.83	0.83	0.83
STTD^8^ P, %	0.44	0.44	0.44	0.44
ME, Mcal/kg	3.23	3.23	3.17	3.17
NE, Mcal/kg	2.45	2.45	2.37	2.37
dUA^9^	343	343	327	327
dEB^10^	428	428	411	411

^1^Treatments include: insoluble fiber without enzyme (IF−); insoluble fiber with enzyme (IF+); soluble fiber without enzyme (SF−); soluble fiber with enzyme (SF+).

^2^Vitamin premix provided the following (per lb of premix) 750,000 IU of vitamin A; 150,000 IU of vitamin D3; 6,000 IU of vitamin E; 300 mg of menadione (to provide vitamin K); 750 mg of riboflavin; 2,500 mg of d-pantothenic acid; 3 mg of vitamin B12, and 4,500 mg of niacin.

^3^Mineral premix provided the following (per lb or premix): 10,000 mg of Fe (ferrous sulfate); 10,000 mg of Zn (zinc sulfate); 3,000 mg of Mn (manganese sulfate); 1,500 mg of Cu (copper sulfate); 27 mg of I (calcium iodate); 27 mg of Se (sodium selenite).

^4^Rovabio Advance P10; Adisseo, Antony, France.

^5^Axtra PHY GOLD; DuPont Nutrition and Biosciences, Copenhagen, Denmark.

^6^Standard ileal digestible.

^7^Total sulfur amino acids (Met + Cys).

^8^Standardized total tract digestible.

^9^Dietary undetermined anion.

^10^Dietary electrolyte balance.

A weekly sample of feed was taken from bins throughout the course of the study, pooled, and stored at −20 °C for comparative analysis. The analyzed nutrient composition of feed ingredients and experimental diets are presented in Tables 2 and 3, respectively. Sows were fed experimental diets from days 28 to 109 of gestation at a feeding level of 2.1 kg/d as-fed to achieve 11 g of SID-Lys and at least 4.5 Mcal of NE per d. The daily ration was provided in a single offering at 0600 hours throughout gestation period. Diets were fortified with 10.2 g of titanium dioxide (**TiO**_**2**_) as an indigestible marker for determining apparent total tract digestibility (**ATTD**) for 8-d prior to fecal and urine collections. During each metabolism period, water allowance was set to 80 mL/kg of BW to mitigate the impact of excess water intake on urine measurements. Otherwise, during the study sows had ad libitum access to water.

**Table 2.** Analyzed nutrient composition of feed ingredients (as-is basis)^1^^,^^2^^,^^3^

	Ingredients				
Item^4^, %	Corn	SBP	DDGS	SBM	CGM
DM	88.2	94.5	89.6	90.9	92.2
TDF	10.1	54.6	38.8	19.4	11.2
IDF	9.7	33.4	37.7	17.6	10.5
SDF	0.4	21.2	1.1	1.8	0.7
NDF	8.5	41.6	34.6	8.2	10.3
ADF	2.0	26.9	11.5	4.5	3.2
CP	7.9	8.6	30.7	49.7	64.3
CF	2.8	< 0.2	5.8	0.6	2.1
Starch	47.9	0.8	7.0	4.9	32.1
GE, Mcal/kg	4.2	4.0	4.9	4.6	5.5
Bulk density g/L	764.4	321.2	591.8	858.1	614.4

^1^A total of 36 confirmed gestating sows balanced by parity were randomly assigned to 1 of 4 diets (*s* = 9) in a 2 × 2 factorial arrangement.

^2^Factors included fiber type of either insoluble fiber (**IF**; 15.1 IDF%) or soluble fiber (**SF**; 4.6 SDF%) and with (**+**) or without (−) enzyme supplementation (Rovabio Advance P10; Adisseo, Antony, France).

^3^Diets were fed from days 28 to 109 of gestation at a feeding level of 2.1 kg/d. On days 50 and 99 of gestation, a 9-d metabolism study was conducted.

^4^DM = dry matter; OM = organic matter; TDF = total dietary fiber; IDF = insoluble dietary fiber; SDF = soluble dietary fiber; NDF = neutral detergent fiber; ADF = acid detergent fiber; ADL = acid detergent lignin; CP = crude protein; CF = crude fat; GE = gross energy.

**Table 3.** Analyzed nutrient composition of experimental diets (as-is basis)^1^^,^^2^^,^^3^

	Diets			
Item^4^, %	IF−	IF+	SF−	SF+
DM	89.5	89.9	89.5	90.8
OM	84.9	85.4	84.8	86.3
TDF	16.5	16.3	16.7	17.0
IDF	15.3	15.0	11.9	12.5
SDF	1.2	1.3	4.8	4.5
NDF	13.6	12.9	15.5	15.6
ADF	4.6	3.9	10.6	13.3
ADL	1.4	1.5	4.2	4.1
Insoluble AX	5.4	5.3	2.8	3.2
Soluble AX	0.5	0.6	1.1	1.2
CP	14.9	15.2	14.9	14.8
Ash,	4.7	4.5	4.6	4.5
CF	3.6	3.9	2.3	2.5
Starch	54.9	51.0	45.4	44.3
Enzyme, U/kg^5^	< 300	3090	< 300	3005
GE, Mcal/kg	4.23	4.26	4.15	4.16
Bulk density g/L	706.3	708.6	605.6	584.0

^1^A total of 36 confirmed gestating sows balanced by parity were randomly assigned to 1 of 4 diets (*n* = 9) in a 2 × 2 factorial arrangement.

^2^Factors included fiber type of either insoluble fiber (**IF**; 15.1 IDF%) or soluble fiber (**SF**; 4.6 SDF%) and with (**+**) or without (−) enzyme supplementation (Rovabio Advance P10; Adisseo, Antony, France).

^3^Diets were fed from days 28 to 109 of gestation at a feeding level of 2.1 kg/d. On days 50 and 99 of gestation, a 9-d metabolism study was conducted.

^4^DM = dry matter; OM = organic matter; TDF = total dietary fiber; IDF = insoluble dietary fiber; SDF = soluble dietary fiber; NDF = neutral detergent fiber; ADF = acid detergent fiber; ADL = acid detergent lignin; CP = crude protein; CF = crude fat; GE = gross energy.

^5^Viscosimetry unit (U/kg) is the amount of enzyme which hydrolyzes the substrate reducing the viscosity of the solution, to give a change in relative fluidity of 1 (dimensionless) unit/min at 300 °C and pH 5.5.

### Sample and data collection

On day 1 of each metabolism period, sows were weighed to determine water allowance, serum and plasma were collected via jugular venipuncture with a 16 gauge × 4-inch needle into a neutral syringe, serum coagulant syringe, and a lithium heparinized syringe (Sarstedt, Nūmbrecht, Germany), and fresh fecal samples were collected using sterile instruments. Serum and plasma were separated by centrifugation at 1,500 × *g* for 15 min at 4 °C, and 1,000 × *g* for 10 min at 4 °C, respectively. Aliquots of each fraction were subsampled and stored at −80 °C for future analysis. Fecal samples were subsampled, flash-frozen in liquid nitrogen, and stored at −80 °C for further analysis.

Sows were provided daily feed allotment at 0600 hours during each metabolism period, allowed 1 h for meal consumption, and any orts remaining were collected and weighed. Prior to feeding on day 4, crates were cleaned of organic matter and collections trays with a separation screen were placed under each crate to allow for separate but total collection of urine and feces for a 96-h period. During this period, fecal samples were collected via grab sampling, weighed, and stored at −20 °C. Urine was collected every 12 h in an acid-washed container with 40 mL 6 *N*-HCl to minimize nitrogen volatilization and the pH of urine was checked. The urine containers were weighed, filtered through glass wool and cheesecloth, subsampled, and stored in acid-washed containers at −20 °C for future analysis.

Following energy balance collections, day 8 was designated for a lactulose-mannitol intestinal permeability study. On the morning of day 8, sows were orally administered a 100 mL solution containing 15 g of suspended lactulose (99.9% purity; Spectrum Chemical Corp, Gardena, CA) and 5 g of mannitol (99.8% purity, Fisher Chemical, Ottawa, ON) and underwent a 24-h total urine collection for the assessment of in vivo small intestinal permeability. Collected urine was weighed, and filtered through cheesecloth, and the total volume was stored at −20 °C for further analysis. Sows were moved back to a gestation stall at the conclusion of the mid gestation metabolism period and moved to a farrowing room after the late-gestation metabolism period.

### Diet, urine, and fecal analytical methods

At the end of each replication, fecal and urine samples were thawed, homogenized within pig and period, and subsampled for further analysis. A convection oven (Sheldon Manufacturing, Cornelius, OR) was used to dry the diet and fecal samples at 60 °C until a constant dry matter (**DM**) weight was attained. Dried samples were ground to 1.0 mm particle size in an upright centrifugal grinding mill. Whatman 41 filter paper (GE Healthcare Life Sciences, Chicago, IL, USA) was used to filter urine subsamples under vacuum before analysis. Diet and fecal samples were analyzed in triplicate for crude fat (**CF**; method 2003.05; AOAC, 2007), neutral detergent fiber (**NDF**) using the Van Soest and Robertson (1979) method, acid detergent fiber (**ADF**), and acid detergent lignin (**ADL**) using the Goering and Van Soest (1970) method. Diets were analyzed for starch in triplicate using a Megazyme total starch assay kit (Wicklow, Ireland; modified method 996.11, AOAC, 2007). Enzyme activity of the diets was determined by a commercial laboratory (Eurofins, Des Moines, IA). Diet, fecal, and urine samples were analyzed for nitrogen (method 990.03; AOAC, 2007; TruMac; LECO Corp., St. Joseph, MI) in duplicate. An ethylenediaminetetraacetate sample (9.56%± 0.08% nitrogen) was used for standard calibration and crude protein (**CP**) was calculated as nitrogen × 6.25. Diets were analyzed in triplicate for soluble arabinoxylans and insoluble arabinoxylans, using a gas chromatography–smass spectrometer in electron impact mode (Agilent Technologies, Palo Alto, CA, USA) according to the methods and conditions described by Englyst et al. (1994). Myo-inositol was used as an internal standard.

Diet and fecal samples were analyzed in duplicate for total dietary fiber (**TDF**) using Ankom TDF Analyzer (Ankom Technology, Macedon, NY, method: 991.43; AOAC 2007). Diet and fecal samples were analyzed in duplicate for DM (method 930.15), ash (method: 942.05), and TiO_2_ using the Fenton and Fenton (1979) method. Diet and fecal samples were analyzed in duplicate for gross energy (**GE**) using a bomb calorimeter (model 6400; Parr Instrument Co., Moline, IL). Benzoic acid (6,31  9 kcal/kg) was used as the standard for calibration. Urine GE determination was conducted using a modified method from Petry et al., (2020), whereas 2 mL of urine was added to 0.2 g of cotton, frozen at −20 °C, and lyophilized for 48 hours (Genesis SQ SuperES-55 Lyophilizer, Stone Ridge, NY). Urine plus cotton-dried samples were analyzed for GE in triplicate. Urinary energy was calculated from the difference between the energy determined in cotton and the energy determined in the samples containing both urine and cotton. A CV threshold of less than 1% was used for DM, TiO_2_, CP, and GE, and less than 5% for NDF, ADF, Starch, and CF.

### Lactulose and mannitol analysis

A 1-mL aliquot of intestinal permeability urine samples were analyzed for lactulose and mannitol concentration at the Texas Tech University Metabolomics Laboratory (Lubbock, TX), as described by Hurum and Rohrer (2016). Samples were diluted 1:100 and analyzed using a Dionex Ultimate 3000 Nano-LC, Vanquish Liquid chromatography system with a Dionex CarboPac MA1 4 × 50 mm guard followed by a CarboPac MA1 BioLC Analytical column (Thermo Scientific, Sunnyvale, CA, USA). Sodium hydroxide was used as the mobile phase (480 mmol/L) at a flow rate of 0.4 mL/min. Standards and negative controls were prepared and analyzed in tandem with urine samples.

### Systemic biomarker analysis

Serum malondialdehyde (**MDA)** was measured with a thiobarbituric acid reactive substances kit using fluorometric procedures (TBARS Assay Kit, Cayman Chemical Company, Ann Arbor, MI). Lipopolysaccharide-binding protein (**LBP**) was measured in lithium heparinized plasma diluted 1:200 with the provided assay buffer using an enzyme-linked immunosorbent assay kit (Hycult Biotech, Plymouth Meeting, PA). Plasma total antioxidant capacity (**TAC)** was measured using a colorimetric assay (Antioxidant Assay Kit, Cayman Chemical Company, Ann Harbor, MI). Immediately after centrifugation, 100 μL of fresh plasma was diluted 1:50 with the provided assay buffer. TAC of plasma was defined as all antioxidants within a sample that inhibited the oxidation of 2,2’-Azino-di-3-ethylbenzthiazoline sulphonate by metmyoglobin relative to a water-soluble tocopherol analog and quantified as millimolar Trolox equivalents as described by Miller et al. (1993). Serum cytokines were measured using a 13-plex Immunoassay targeting IFNy, IL-10, IL-12, IL-18, IL-1α, IL-1β, IL-4, IL-8, TNF-α, GM-CSF, IL-1ra, and IL-2 (Eve Technologies, Calgary, AB). A CV threshold of less than 5% was used for all biomarker analyses.

### Calculations and statistical analysis

The ATTD of DM, OM, GE, CP, TDF, SDF, IDF, NDF, ADF, CF, and hemicellulose was calculated using the index method as described by Oresanya et al. (2007):


$$\begin{array}{llll} ATTD\;\%= & \{100\;[100\times \left(\%\;TiO_{2}in\;feed/\;\%\;TiO_{2}in\;feces\right)\;\times \; & (concentration\;of\;component\;in\;feces/\; & concentration\;of\;component\;in\;feed)]\}. \end{array}$$

Digestible energy (**DE**) was calculated using the total tract digestibility coefficient for GE, ME was computed by subtracting urinary energy from DE. Methane losses were omitted from ME estimates. Net energy (**NE**) was estimated from ME using an equation described by Noblet (1994).

Data were analyzed according to the following linear mixed model:


$$\begin{array}{lll} Y_{ijkl}=\; & \mu +\tau_{i\;}+\upsilon_{j}+\rho_{k\;}+\;\tau_{i\;}\upsilon_{j}+\tau_{i\;}\rho_{k\;}+\; & \upsilon_{j}\rho_{k\;}+\tau_{i\;}\upsilon_{j}\rho_{k\;}+\kappa_{l}+e_{ijklm} \end{array}$$

Where $Y_{ijkl}$ is the observed value for a given sow within the *i*th level of fiber, *j*th level of enzyme of the *k*th period; μ is the general mean; $\tau_{i\;\;\;}$ is the fixed effect of the *i*th fiber (*i* = 1 to 2); ${\;\upsilon \;}_{j}$ is the fixed effect of the *j*th enzyme (*j* = No or Yes); $\rho_{k\;}$ is the fixed effect of the *k*th period (*k* = 1 to 2); $\tau_{i\;\;\;}{\;\upsilon \;}_{j}$ is the interaction term of Fiber × Enzyme; $\tau_{i\;}\rho_{k\;}$ is the interaction term of Fiber ×    Period; $\upsilon_{j}\rho_{k\;}$ is the interaction term of Enzyme × Period; $\tau_{i\;}\upsilon_{j}\rho_{k\;}$ is the interaction term of Fiber ×    Enzyme × Period; $\kappa_{l}$ is the random effect of parity; and $e_{ijklm}$ is the associated variance as described by the model for $Y_{ijkl}$ (l = 1 through 36); assuming $e_{ijklm}\;\;\;∼\;N(0,\;I\;\sigma_{e}^{2}$), where *I* is the identity matrix.

The PROC UNIVARIATE procedure in SAS 9.4 (SAS Inst., Cary, NC) was used to verify the normality and homogeneity of the studentized residuals. The model was analyzed using PROC MIXED as described. Least square means were separated using Fisher’s Least Significant Difference test, and treatment differences were considered significant if P ≤ 0.05 and trends if 0.05 > *P* ≤ 0.10.

## Results

All sows allotted to treatment completed the experiment, and no treatments were administered during the experimental period. No interaction between main effects and replicas was observed for the reported dependent variables. Thus, replicate was analyzed as a random effect within the model to account for replicate associated covariance.

### Nutrient and energy digestibility

At mid gestation, sows fed SF tended to have 1.5% greater DM digestibility than IF, but at late gestation IF and SF tended to have increased ATTD of DM by 2.8% and 1.7%, relative to mid gestation, respectively, (Period × Fiber *P* = 0.096; Table 4). Compared to mid gestation, IF had greater ATTD of GE, TDF, NDF, and ADF digestibility by 2.8%, 9.8%, 12.8%, and 12.7% in late gestation, respectively (Period × Fiber *P* < 0.05). Similarly, sows fed SF in late gestation had increased GE and NDF digestibility by 1.1% and 3.7%, respectively (Period × Fiber *P* < 0.05), but at mid gestation sows fed SF did not differ from IF. The ATTD of TDF was greater in sows fed SF at mid gestation but did not differ from IF at late gestation (Period × Fiber *P* = 0.014). Regardless of point in gestation or fiber type, the supplementation of multicarbohydrases increased DM, GE, NDF, and TDF digestibility by 2.3%, 2.0%, 8.0%, and 6.5%, respectively (Enzyme *P* < 0.05).

**Table 4.** The influence of period × fiber type and main effect of enzyme on ATTD of DM, GE, TDF, and NDF in gestating sows^1^^,^^2^

Item	Mid gestation^3^		Late gestation		Enzyme			P-value	
	IF	SF	IF	SF	−	+	SEM^5^	P × F	Enzyme
ATTD^4^, %									
DM	86.8^x^	88.1^y^	89.3^z^	89.6^z^	87.4	89.4	0.56	0.096	<0.001
GE	86.4^a^	88.1^bc^	88.9^c^	89.1^d^	87.2	89.0	0.58	0.019	<0.001
NDF	66.3^a^	74.1^b^	74.8^bc^	76.9^c^	70.2	75.8	1.41	0.002	0.029
TDF	65.1^a^	68.6^b^	71.5^c^	72.1^c^	67.3	71.7	1.12	0.014	<0.001

^a,b,c,d^Within a row, treatment means without a common superscript differ as significant, *P *< 0.05.

^x,y,z^Within a row, treatment means without a common superscript differ as a tendency, 0.05 ≥ *P* ≤ 0.100.

^1^A total of 36 confirmed gestating sows were randomly assigned to 1 of 4 diets (n = 9) in a 2 × 2 factorial arrangement.

^2^Factors included fiber type of either insoluble fiber (**IF**; 15.1 IDF%) or soluble fiber (**SF**; 4.6 SDF%) and with (**+**) or without (**−**) enzyme supplementation (Rovabio Advance P10; Adisseo, Antony, France).

^3^Diets were fed from days 28 to 109 of gestation at a feeding level of 2.1 kg/d. On day 50 and 99 of gestation, a 9-d metabolism study was conducted.

^4^Apparent Total Tract Digestibility (ATTD)% = {100 − [100 × (% TiO_2_ in feed/ % TiO_2_ in feces) × (concentration of component in feces/ concentration of component in feed)]}.

^5^Pooled SEM.

**Figure 1.**
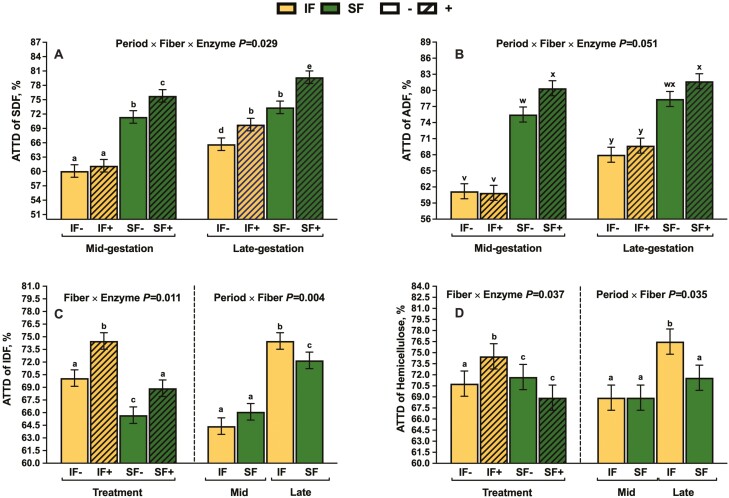
The influence of period × fiber × enzyme interaction on ATTD of ADF (**A**) and SDF (**B**) and the influence of fiber × enzyme interaction with the period × fiber type interaction on ATTD of IDF (**C**) hemicellulose (**D**) in gestating sows.

There was a period × fiber × enzyme interaction for the ATTD of SDF and a tendency was observed for ADF (Figure 1A and B, respectively). Whereas sows fed SF+ had the greatest ATTD of SDF in late gestation, the ATTD of SDF did differ at mid or late gestation for SF−. There was no influence of carbohydrase supplementation on the ATTD of SDF at mid gestation in IF, but IF+ was 8.1% greater than IF− at late gestation. Irrespective of collection period, IF with multicarbohydrase supplementation had 5.2% greater hemicellulose digestibility, compared to IF− (Fiber × Enzyme *P* = 0.037; Figure 1D). Relative to SF or IF at mid gestation, sows fed IF in late gestation had increased hemicellulose digestibility by 11% (Period × Fiber *P* = 0.035). Sows fed SF, with multicarbohydrase supplementation, and late gestation had increased ATTD of OM by 0.8%, 1.2%, and 1.6%, respectively (Fiber, Enzyme, and Period *P* < 0.022). Similarly, sows fed insoluble fiber, with multicarbohydrase supplementation, and late gestation had increased ATTD of CF by 3%, 2.6%, and 1.9%, respectively (Fiber, Enzyme, and Period *P* < 0.049).

### Energy balance

Energy intake tended to be lower in sows fed SF (*P* = 0.072; Table 5), but did not differ for time or enzyme due to equivalent feed intake levels set in the experimental design. Compared to mid gestation, sows in late gestation fed SF or supplemented with a multicarbohydrase excreted less total GE by 10.4%, 9.4%, and 14.2%, respectively (fiber, enzyme, and period *P* < 0.001). Relative to IF, no multicarbohydrase, and at mid gestation, total fecal energy excretion decreased in sows fed SF, multicarbohydrase supplementation, and at late gestation by 7.8%, 9.6%, and 13.0%, respectively (fiber, enzyme, and period *P* < 0.009). Similarly, compared to their counterparts, sows fed SF, supplemented with enzymes, and at late gestation had decreased urinary energy excretion in by 14.4%, 9.6%, and 15.7%, respectively (Fiber, enzyme, and period *P* < 0.049). Total DE intake increased with multicarbohydrase supplementation and at late gestation by 2.1% and 1.9%, respectively (enzyme, period *P* < 0.001). Likewise, daily ME intake increased with multicarbohydrase addition and at late gestation by 3.2% and 3.4%, respectively, (Enzyme, Period *P* < 0.001). Similarly, DE of DMI increased in multicarbohydrase product supplementation and in late gestation by 3.6% and 2.0%, respectively (Enzyme, Period *P* < 0.001). In addition, ME of DMI increased in multicarbohydrase product supplementation and in late gestation by 2.8% and 3.5%, respectively (Enzyme, Period *P* < 0.041).

**Table 5.** The influence fiber type, enzyme supplementation, and collection period of main effects on energy balance, GE, DE, ME, and ATTD of OM and CF in gestating sows^1^^,^^2^

Item	Fiber		Enzyme		Period^3^			P-value		
	IF	SF	−	+	Mid	Late	SEM^4^	Fiber	Enzyme	Period
Energy Balance, GE kcal/d										
Intake	7950	7860	7898	7931	7897	7904	12.1	0.072	0.641	0.918
Total Excreted	1514	1356	1506	1364	1545	1325	46.4	<0.001	<0.001	<0.001
Fecal	981	904	985	900	1008	877	20.4	0.009	0.003	<0.001
Urinary	533	456	519	469	536	452	36.6	0.049	0.033	0.002
Energy Intake kcal/d										
GE	7950	7860	7898	7931	7897	7904	12.1	0.072	0.641	0.918
DE	6968	6946	6884	7030	6891	7022	23.3	0.446	<0.001	<0.001
ME	6434	6494	6362	6565	6354	6573	49.7	0.119	<0.001	<0.001
Energy kcal/kg of DMI										
GE	4341	4249	4318	4267	4290	4294	-	-	-	-
DE	3806	3751	3723	3856	3740	3815	21.8	0.201	<0.001	<0.001
ME	3511	3510	3482	3583	3451	3571	31.5	0.986	0.041	<0.001
ME:DE Ratio	92	93	92	93	92	94	0.71	0.433	0.118	0.011
ATTD^5^, %										
OM	90.59	91.32	90.43	91.47	90.22	91.68	0.22	0.022	0.001	<0.001
CF	92.77	90.08	90.23	92.61	90.58	92.27	0.60	< 0.001	< 0.001	0.049

^1^A total of 36 confirmed gestating sows blocked by parity were randomly assigned to 1 of 4 diets (*n* = 9) in a 2 × 2 factorial arrangement.

^2^Factors included fiber type of either insoluble fiber (IF; 15.1 IDF%) or soluble fiber (SF; 4.6 SDF%) and with (+) or without (−) enzyme supplementation (Rovabio Advance P10; Adisseo, Antony, France).

^3^Diets were fed from days 28 to 109 of gestation at a feeding level of 2.1 kg/d. On days 50 and 99 of gestation, a 9-d metabolism study was conducted.

^4^Apparent total tract digestibility (ATTD)% = {100 − [100 × (% TiO_2_ in feed/ % TiO_2_ in feces) × (concentration of component in feces/ concentration of component in feed)]}.

^5^Pooled SEM.

### Nitrogen balance

Based on the design, N intake did not differ (*P* > 0.05). Sows in late gestation excreted 13.2% less total nitrogen compared to sows in mid gestation (Period *P* < 0.001; **Table 6**). Multicarbohydrase supplementation decreased fecal N excretion by 11.9% (Enzyme *P* = 0.034). In late gestation, sows had a 13% decrease in fecal N excretion compared to mid gestation (Period *P* = 0.001). Sows in late gestation excreted 12.3% less N in urine compared to mid gestation (Period *P* = 0.003). Multicarbohydrase supplementation, multicarbohydrase supplementation increased retained nitrogen on a grams per day basis by 12.2% (Enzyme *P* = 0.04). Relative to mid gestation, sows in late gestation also increased retained nitrogen on a grams per day basis by 21.2% (Period *P* < 0.001). Compared to sows in mid gestation and without multicarbohydrase supplementation, sows in late gestation with multicarbohydrase supplementation increased ATTD of N by 1.8% and 2.5%, respectively (Enzyme, Period *P* = 0.001).

**Table 6.** The impact of main effects of fiber type, enzyme supplementation, and collection period on N balance and ATTD of N in gestating sows^1^^,^^2^

Item	Fiber		Enzyme		Period^3^			P-value		
	IF	SF	−	+	Mid	Late	SEM^4^	Fiber	Enzyme	Period
N balance, g/d										
Intake	44	44	44	44	44	44		0.772	0.668	0.924
Total excreted	25.5	25.5	25.9	24.9	27.3	23.7	0.77	0.965	0.061	<0.001
Fecal	6.5	6.4	6.7	5.9	6.9	6.0	0.23	0.595	0.034	0.001
Urinary	19.0	19.1	19.1	19.0	20.3	17.8	0.76	0.940	0.951	0.003
N retained, g/d	18.5	18.0	17.2	19.3	16.5	20.0	0.77	0.650	0.04	<0.001
N retained as % of intake	41.9	41.4	40.1	43.3	37.7	45.7	1.65	0.815	0.069	<0.001
ATTD of N, %	85.2	85.3	84.5	86.0	84.2	86.3	0.34	0.912	0.001	0.001

^1^A total of 36 confirmed gestating sows balanced by parity were randomly assigned to 1 of 4 diets (*n* = 9) in a 2 × 2 factorial arrangement.

^2^Factors included fiber type of either insoluble fiber (IF; 15.1 IDF%) or soluble fiber (SF; 4.6 SDF%) and with (**+**) or without (**−**) enzyme supplementation (Rovabio Advance P10; Adisseo, Antony, France).

^3^Diets were fed from days 28 to 109 of gestation at a feeding level of 2.1 kg/d. On days 50 and 99 of gestation, a 9-d metabolism study was conducted.

^4^Pooled SEM.

### Markers of gut barrier integrity, immune function, inflammation, and oxidative status

**Figure 2.**
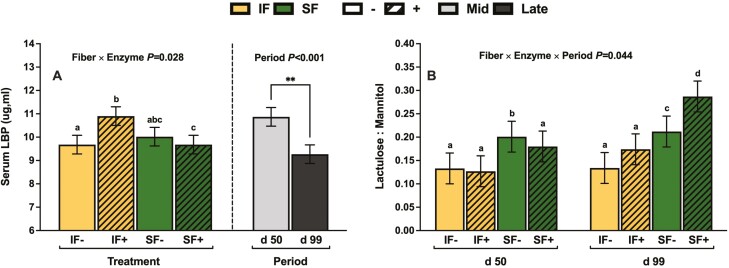
The influence of fiber × enzyme interaction and period on circulating serum LBP (**A**) and the fiber × enzyme × period interaction on lactulose: mannitol recovery (**B**) in gestating sows.

Sows fed IF tended to have decreased circulation of IL-10 by 42.1% compared to sows fed SF (Fiber *P* = 0.096; Table 7). In comparison to no enzyme, multicarbohydrase supplementation decreased the presence of circulating IL-4, IL-8, and TNFα by 46.2%, 58.3%, and 52.4%, respectively (Enzyme *P* < 0.048). Sows fed IF hve decreased circulation of IL6 by 53.6% compared to SF (Fiber *P* = 0.032). Sows in mid gestation had decreased levels of TAC and MDA relative to sows in late gestation by 11.8% and 26.5%, respectively (Period *P* < 0.015). The supplementation of multicarbohydrase product increased GM-CSF in IF by 48.8% but decreased in SF by 52.7% (Fiber × Enzyme *P = *0.042; Table 8). Sows fed SF+ had increased IL-1ra by 55.4% (Fiber × Enzyme *P = *0.035). Similarly, sows fed SF+ had increased IL-2 by 76.2% (Fiber × Enzyme *P = *0.042). Sows fed IF+ had increased circulating LBP by 12.6% (Fiber × Enzyme *P = *0.028; **Figure 2A**). The response of SF− and SF+ across gestation drove a 3-way interaction for lactulose to mannitol ratio (Fiber × Enzyme × Period *P* = 0.044; **Figure 2B**). Whereas, sows fed SF− at mid gestation had an 11.7% increase in lactulose to mannitol ratio compared to SF+; conversely, in late gestation, SF+ had a 35.4% increase in lactulose to mannitol ratio compared to SF−.

**Table 7.** The main effect of fiber type, enzyme supplementation, and collection period on systemic biomarkers of immune function, inflammation, and oxidative status in gestating sows^1^^,^^2^

Item	Fiber		Enzyme		Period		SEM^5^	P-value		
	IF	SF	−	+	day 50	D 99		Fiber^3^	Enzyme^3^	Period^4^
Cytokines, ng/mL										
IFNy	25.08	16.13	14.86	26.35	19.81	21.39	8.97	0.228	0.124	0.829
IL-10	4.97	8.59	6.37	7.19	6.07	7.49	2.93	0.096	0.768	0.609
IL-12	1.30	1.87	1.43	1.75	1.53	1.65	0.48	0.231	0.506	0.797
IL-18	9.57	10.15	9.37	10.35	11.16	8.56	2.74	0.797	0.668	0.254
IL-1α	0.20	0.25	0.20	0.25	0.21	0.24	0.08	0.428	0.408	0.604
IL-1β	2.18	2.92	2.19	2.91	2.44	2.66	0.82	0.403	0.427	0.797
IL-4	10.60	12.03	17.97	9.67	10.45	14.19	3.59	0.782	0.012	0.270
IL-6	0.65	1.40	1.14	1.01	0.83	1.52	0.23	0.032	0.834	0.071
IL-8	0.16	0.19	0.24	0.10	0.15	0.20	0.06	0.633	0.048	0.569
TNFα	0.86	0.99	1.26	0.60	0.91	0.95	0.24	0.723	0.022	0.910
Oxidative status										
TAC^6^, mM	6.28	6.12	6.27	6.13	5.81	6.59	0.87	0.591	0.626	0.008
MDA^7^,μmol/μL	6.72	6.34	6.39	6.67	5.53	7.52	0.56	0.638	0.724	0.015

^1^No significant interactions observed among enzyme supplementation, fiber type or collection period (*P* > 0.10).

^2^A total of 36 confirmed gestating sows blocked by parity were randomly assigned to 1 of 4 diets (*n* = 9) in a 2 × 2 factorial arrangement.

^3^Factors included fiber type of either insoluble fiber (IF; 15.1 IDF%) or soluble fiber (SF; 4.6 SDF%) and with (+) or without (−) enzyme supplementation (Rovabio Advance P10; Adisseo, Antony, France).

^4^Diets were fed from days 28 to 109 of gestation at a feeding level of 2.1 kg/d. On days 50 and 99 of gestation serum was collected.

^5^Pooled SE.

^6^Total antioxidant capacity.

^7^Maondealdehyde.

**Table 8.** The simple effects^1^ of fiber type by enzyme supplementation interaction on serum cytokines and in gestating sows^2^ at 2 collection periods

Serum	Treatment^4^				Period			P-value	
	IF−	IF+	SF−	SF+	day 50	day 99	SEM^5^	F × E	Period
Cytokine, ng/mL									
GM-CSF^6^	0.22^a^	0.43^b^	0.55^b^	0.26^a^	0.36	0.36	0.047	0.042	0.997
IL-1ra	2.12^a^	2.20^a^	2.69^a^	4.18^b^	2.48	3.11	0.501	0.035	0.579
IL-2	1.53^a^	1.67^a^	1.64^a^	2.89^b^	1.81	2.06	0.402	0.042	0.707

^a,b,c^Within a row and effect, treatment means without a common superscript differ, *P* < 0.05.

^1^No significant interactions observed among enzyme × fiber × period (*P* > 0.10).

^2^A total of 36 confirmed gestating sows blocked by parity were randomly assigned to 1 of 4 diets (*n* = 9) in a 2 × 2 factorial arrangement.

^3^Factors included fiber type of either insoluble fiber (**IF**; 15.1 IDF%) or soluble fiber (**SF**; 4.6 SDF%) and with (**+**) or without (**−**) enzyme supplementation (Rovabio Advance P10; Adisseo, Antony, France).

^4^Diets were fed from days 28 to 109 of gestation at a feeding level of 2.1 kg/d. On days 50 and 99 of gestation serum was collected.

^5^Pooled SEM.

^6^Granulocyte macrophage colony-stimulating factor.

## Discussion

DF is commonly included in sow diets for its distinct properties of bulk density, satiety, and weight management (Agyekum and Nyachoti, 2017; Huang et al., 2020). However, DF is a complex nutrient comprised of a surfeit of polysaccharides with chemical and physical properties that influence gastrointestinal physiology (Knudsen, 2001). Corn-based DF is poorly fermented due to its insolubility and complex branched structure; this alters the rate of passage of digesta and decreases nutrient and energy digestibility (Gutierrez et al., 2013). Conversely, soluble DF sources, such as SBP, have been shown to benefit fermentability, gut health, and immune regulation in pigs (Li et al., 2019a; Shang et al., 2021). Supplementing exogenous carbohydrases is a potential strategy to improve fiber utilization and increase the beneficial aspects of poorly fermented fiber. Intriguingly, improvements in oxidative status, inflammatory responses, and intestinal permeability in the weaned and grower pig have been observed in the presence of both DF and carbohydrases (Li et al., 2019a, b; Petry et al., 2020). However, there is a paucity of research on multicarbohydrase supplementation in IF and SF in sow diets, and thus, is the focus of this study.

Supplementing a multicarbohydrase in gestating sow diets herein increased the ATTD of DM, GE, TDF, NDF, OM, CF, and N, irrespective of DF type. This aligns with an abundance of literature on the efficaciousness of carbohydrases to increase digestibility in both weaned and grow-finish pigs outlined by Torres-Pitarch et al., (2017 and 2019). The primary mechanism of a carbohydrase is to hydrolyze polysaccharides into metabolizable and fermentable substrates. The interactions between enzyme supplementation and fiber type indicated the distinctiveness in carbohydrase responses across targeted fiber types in SF and IF. The improved ATTD of SDF and ADF within SF+ diets could be attributed to the heightened breakdown of pectin and cellulose, primary fiber components in SBP (Navarro et al., 2018). In contrast, the increased ATTD of IDF and hemicellulose within IF+ was likely due to the enhancement of arabinoxylan hydrolysis (Chen et al., 2020). The elevated cellulose and pectin substrates in SF likely enhance cellulase and pectinase efficiency of the multicarbohydrase (Fahey et al., 2019). Meanwhile, xylanase and debranching enzymes may selectively target arabinoxylan in IF. Additionally, hydrolyzing multiple polysaccharides could improve the bioavailability of entrapped nutrients, potentially increasing OM, CF, and N digestibility (Zeng et al., 2018).

A period by fiber interaction was observed for the ATTD of GE and NDF, whereby sows in late gestation had increased digestibility regardless of fiber type. A similar effect was observed by Feyera et al., (2021) and could be explained by altered digestion kinetics in late gestation resulting in improved digestive efficiency (Bradley et al., 2007). Interestingly, there is a period by fiber response observed for the ATTD of hemicellulose; whereas sows fed IF had greater digestibility in late gestation, compared to SF. This is likely a result of increased capacity to ferment arabinoxylan, the predominate hemicellulose found in corn DDGS, with increased microbial adaptation time and reduced rate of passage in late gestation (Bradley et al., 2007; Jaworski et al., 2015).

It is well established that there is an inverse relationship between fiber inclusion and energy digestibility in swine, but carbohydrases show promise of improving energy bioavailability (Kerr and Shurson, 2013; Petry et al., 2020). The effects of fiber, enzyme, and period were observed for total excreted, fecal, and urinary GE. The effect of fiber on fecal GE excretion is in alignment with the poor fermentability and increased rate of passage associated with corn-based DF (Knudsen, 2001; Acosta et al., 2020) and conversely, the increase in fermentation of SBP, which has been observed in sows (Renteria-Flores et al., 2008). As gestation progresses, sows have increased maintenance energy demands, but a decreased rate of passage of digesta resulting in improved nutrient uptake (Noblet et al., 1990; Bradley et al., 2007). The period effect on urine and fecal GE excretion supports a greater efficiency in digesting and metabolizing energy as gestation progresses also observed by Renteria-Flores et al., (2008). Increased metabolic efficiency at the end of gestation has been associated with alterations in metabolic trajectory due to homeorhetic control mechanisms (Bauman and Currie, 1980). However, it should be noted these improvements in adaptive efficiencies cannot be separated from an increase in total BW (Noblet et al., 1990).

The supplementation of a multicarbohydrase improved both DE and ME in the gestating sow, irrespective of fiber type. This is congruent with the above discussion on the improvement in ATTD of GE, and in xylanase and cellulase literature in growing pigs (Tsai et al., 2017; Petry et al., 2020). Carbohydrases breaking down complex DF into more accessible oligosaccharides in the large intestine could contribute to SCFA production through microbial fermentation (Williams et al., 2017). The latter would ultimately improve DE value of the diet, and depending on the metabolic efficiencies of the SCFA produced, plausibly ME. The effect of enzyme on ME and urine GE excretion on the surface would suggest a reduction in urine N since urine GE is largely dependent on urine N from urea according to Kerr and Easter (1995). However, there was no impact of enzyme supplementation on urine N output. One explanation of this is beneficial bacteria produced through the fermentation of fiber to SCFAs cause a shift from N excreted as urea in urine to feces (Zervas and Zijlstra, 2002). Alternatively, enzyme supplementation could decrease the abundance of non-nitrogen energy-containing metabolites such as xylitol if pentose fermentation was favored (Huntley et al., 2018).

There was no effect of fiber on N balance because of the iso-nitrogenous formulation of dietary treatments, but sows fed enzyme and later in gestation retained more N. The effect of the enzyme is likely a result of the protease within the additive increasing protein digestibility in the small intestine (Cowieson and Roos, 2013). Likewise, Oryschak et al., (2002) reported a reduction of fecal N excretion in grow-finish pigs supplemented by a carbohydrase cocktail containing β-glucanase and xylanase. The period effect may partially be explained by the late gestating sow partitioning more N towards fetal growth and development (NRC, 2012). An increase in fetal growth and development in the last third of gestation triggers a shift in N portioning to fetal development (McPherson et al., 2004; Mohen et al., 2012). The increase in SID Lysine and estimated ME intake according to the NRC further illustrates the observed effect of the period on total excreted N and N retained in this study. Samuel et al., (2012) support this explanation by observing an increase in daily lysine requirement increasing from early to late gestation.

Oxidative status, inflammation, and gut barrier integrity play a pivotal role in the overall health and longevity of the gestating sow. The increase in circulating serum TAC and MDA in late gestating sows is congruent to what was observed by Berchieri-Ronchi et al., (2011). This alteration in oxidative status may be associated with increased reactive oxygen species due to fetal growth in late gestation and the lipid peroxidation by free radicals (Gaweł et al., 2004). The function of endometrial endothelial cells within the placenta can also be damaged by fetal-induced lipid and protein oxidation, and to combat this, antioxidant enzyme production may be increased systemically (Serdar et al., 2003).

Interleukin-8 and TNFα were both decreased with carbohydrase supplementation and are in alignment with Li et al., (2019) where an enzyme blend of cellulase, β-glucanase, and xylanase decreased pro-inflammatory cytokines through potential suppression of systemic inflammation in the weaned pig. In the gestating sow, TNFαcan potentially be crucial for mitigating systemic inflammatory pressures. A similar theory can be inferred for the IL-4 response, but this cytokine has yet to be successfully evaluated in swine under these conditions. Interestingly, an interaction between fiber and enzyme was observed for GM-CSF, whereas sows fed IF+ had increased levels of GM-CSF, but inversely decreased in sows fed SF+. While the precise mechanisms for these immune modulations are unclear, one potential explanation is the oligosaccharides released in situ by these carbohydrases act in a prebiotic manner (González-Ortiz et al., 2019). Carbohydrase-derived prebiotics in the small intestine of pigs are known to modulate beneficial bacteria whose metabolites can mediate the NF-kB pathway and NLRP3 inflammasome pathway, reducing subsequent cytokine cascades (Bach Knudsen et al., 2018; Petry et al., 2021a, b). Potentially, microbial communities modulated by IF+ decreased NF-kB activation of GM-CSF via T-cells or macrophages, and inversely promoted it in SF+, but further research is needed (Hamilton, 2019).

Circulating serum LBP increased in sows fed IF+ and was decreased in late gestation. In late gestation, He et al., (2019) also observed a decrease in serum LBP in sows under normal thermoregulation, but collectively there is limited literature on the relationship of carbohydrases to LBP. An inflammatory response to lipopolysaccharide is mediated by LBP as it functions as an acute-phase protein to initiate an immune response though pro-inflammatory mediators (Meng et al., 2021). In humans, insoluble DF from whole grains can mediated effects of LBP though sequestering LPS producing bacteria (Seethaler et al., 2022). Plausibly degrading the DF through carbohydrase supplementation may increase lipopolysaccharide exposure in the gastrointestinal tract, but further research is warranted.

The prebiotic mechanism of carbohydrases has been associated with improved gut barrier integrity in weaned and growing pigs due to modulation of the tight junction proteins that span the paracellular space (Tiwari et al., 2018; Long et al., 2021). To our knowledge, this is the first lactulose-mannitol study evaluating intestinal permeability in a sow model but has been done routinely in the weaned and grower pig. Human studies use lactulose-mannitol recovery tests to understand transcellular and paracellular transportation of molecules (Mishra and Makharia, 2012). This study showed an increase in intestinal permeability in sows fed SF+ at mid gestation but decreased at late gestation. The exact reason for this fiber by enzyme by period effect remains unclear. One potential justification is the oligosaccharides that contribute to improved gut barrier integrity via beneficial microbial modulation, are fermented more rapidly in the upper gastrointestinal tract in late gestation due to decreased rate of passage. Thus, limiting their protective effects on intestinal permeability in the distal small intestine.

Concluding, these data indicate that the supplementation of a multicarbohydrase product improves nutrient digestibility and ME in sows fed insoluble and SF. Additionally, multicarbohydrase supplementation improves pro-inflammatory cytokines, alters intestinal permeability, and may reduce systemic inflammatory pressures throughout gestation irrespective of fiber type. However, the mechanisms of action are unique to DF properties and composition.

## Abbreviations

% TiO2: percentage of titanium dioxide
ADF: acid detergent fiber
ATTD: apparent total tract digestibility
BW: body weight
CP: crude protein
CV: coefficient of variation
DE: digestible energy
DF: dietary fiber
DM: dry matter
GE: gross energy
IDF: insoluble dietary fiber
IF: insoluble fiber
IF+: insoluble fiber diet with enzyme
IF−: insoluble fiber diet without enzyme
Mcal: megacalories
ME: metabolizable energy
μ: general mean
NE: net energy
NDF: neutral detergent fiber
SF: soluble fiber
SF+: soluble fiber diet with enzyme
SF−: soluble fiber diet without enzyme
SDF: soluble dietary fiber
SBM: soybean meal
SBP: sugar beet pulp
SID-Lys: standardized ileal digestible lysine
TAC: total antioxidant capacity
TDF: total dietary fiber
τ: fixed effect
ρ: fixed effect of period
υ: fixed effect of enzyme
κ: random effect of parity
e: associated variance

## References

[CIT0001] Acosta, J. A., H. H. Stein, and J. F. Patience. 2020. Impact of increasing the levels of insoluble fiber and on the method of diet formulation measures of energy and nutrient digestibility in growing pigs. J. Anim. Sci. 98:skaa130. doi:10.1093/jas/skaa130 32315034 PMC7275632

[CIT0002] Agyekum, A. K., and C. M. Nyachoti. 2017. Nutritional and metabolic consequences of feeding high-fiber diets to swine: a review. Engineering. 3:716–725. doi:10.1016/j.eng.2017.03.010

[CIT0003] AOAC. 2007. Official methods of analysis of AOAC International. 18th ed. Gaithersburg. MD: AOAC Int.

[CIT0004] Bach Knudsen, K. E., H. N. Lærke, M. S. Hedemann, T. S. Nielsen, A. K. Ingerslev, and G. Nielsen. 2018. Impact of diet-modulated butyrate production on intestinal barrier function and inflammation. Nutrients 10:1499. doi:10.3390/nu10101499 30322146 PMC6213552

[CIT0005] Bauman, D. E., and W. B. Currie. 1980. Partitioning of nutrients during pregnancy and lactation: a review of mechanisms involving homeostasis and homeorhesis. J. Dairy Sci. 63:1514–1529. doi:10.3168/jds.s0022-0302(80)83111-0 7000867

[CIT0006] Bedford, M. R. 2018. The evolution and application of enzymes in the animal feed industry: the role of data interpretation. Brit. Poult. Sci. 59:5. doi:10.1080/00071668.2018.1484074 29877713

[CIT0007] Berchieri-Ronchi, C. B., S. W. Kim, Y. Zhao, C. R. Correa, K. J. Yeum, and A. L. Ferreira. 2011. Oxidative stress status of highly prolific sows during gestation and lactation. Animal. 5:1774–1779. doi:10.1017/S1751731111000772 22440418

[CIT0008] Bradley, C. S., C. M. Kennedy, A. M. Turcea, S. S. Rao, and I. E. Nygaard. 2007. Constipation in pregnancy: prevalence, symptoms, and risk factors. Obstet Gynecol. 110:1351–1357. doi:10.1097/01.AOG.0000295723.94624.b1 18055731

[CIT0009] Chen, H., S. Zhang, and S. W. Kim. 2020. Effects of supplemental xylanase on health of the small intestine in nursery pigs fed diets with corn distillers’ dried grains with solubles. J. Anim. Sci. 98:skaa185. doi:10.1093/jas/skaa185 32497179 PMC7447918

[CIT0010] Cowieson, A. J., and F. F. Roos. 2013. Bioefficacy of a mono-component protease in the diets of pigs and poultry: a meta-analysis of effect on ileal amino acid digestibility. J. Appl. Anim. Nutr. 2:e13. doi:10.1017/jan.2014.5

[CIT0011] Cozannet, P., M. Kidd, N. Yacoubi, P. A. Geraert, and A. Preynat. 2019. Dietary energy and amino acid enhancement from a multi-enzyme preparation. J. Appl. Poult. Res. 28:136–144. doi:10.3382/japr/pfy056

[CIT0061] Englyst, H. N., M. E. Quigley and G. J. Hudson. 1994. Determination of dietary fibre as nonstarch polysaccharides with gas–liquid chromatographic, high-performance liquid chromatographic or spectrophotometric measurement of constituent sugars. Analyst 119: 1497-1509. doi:10.1039/AN9941901497. 7943740

[CIT0012] Fahey, G. C., L. Novotny, B. Layton, and D. R. Mertens. 2019. Critical factors in determining fiber content of feeds and foods and their ingredients. J. AOAC Int. 102:52–62. doi:10.5740/jaoacint.18-0067 30071919

[CIT0013] FASS. 2010. Guide for the care and use of agricultural animals in research and teaching. Third Edition. Champaign, IL: Federation of Animal Science Societies; p. 169

[CIT0014] Fenton, T. W., and M. Fenton. 1979. An improved procedure for the determination of chromic oxide in feed and feces. Can. J. Anim. Sci. 59:631–634. doi:10.4141/cjas79- 081

[CIT0015] Feyera, T., L. Hu, M. Eskildsen, T. S. Bruun, and P. K. Theil. 2021. Impact of four fiber-rich supplements on nutrient digestibility, colostrum production, and farrowing performance in sows. J. Anim. Sci. 99:skab247. doi:10.1093/jas/skab247 34420055 PMC8438544

[CIT0016] Gaweł, S., M. Wardas, E. Niedworok, and P. Wardas. 2004. Malondialdehyde (MDA) as a lipid peroxidation marker. Wiad. Lek.s 57:453–455. 15765761

[CIT0059] Goering, H. K., and P. J. Van Soest. 1970. Forage fiber analyses (Apparatus, reagents, procedures, and some applications). Agric. Handb. Agricultural Research Service, USDA.

[CIT0017] González-Ortiz G. , Gomes G.A., Dos Santos T.T., Bedford M.R., 2019. New strategies influencing gut functionality and animal performance. In: Gonzalez-Ortiz G, Bedford MR, Bach Knudsen KE, Courtin C, Classen HL, editors. The value of fibre. Engaging the second brain for animal nutrition. Wageningen: Wageningen Academic Press; p. 233–253.

[CIT0018] Gutierrez, N. A., B. J. Kerr, and J. F. Patience. 2013. Effect of insoluble-low fermentable fiber from corn-ethanol distillation origin on energy, fiber, and amino acid digestibility, hindgut degradability of fiber, and growth performance of pigs. J. Anim. Sci. 91:5314–5325. doi:10.2527/jas.2013-6328 24045479

[CIT0019] Hamilton, J. A. 2019. GM-CSF-dependent inflammatory pathways. Front. Immunol. 10:2043–2055. doi:10.3389/fimmu.2019.02055 31552022 PMC6737278

[CIT0020] He, J., H. Guo, W. Zheng, Y. Xue, R. Zhao, and W. Yao. 2019. Heat stress affects fecal microbial and metabolic alterations of primiparous sows during late gestation. J. Anim. Sci. Biotechnol. 10:1–12. doi:10.1186/s40104-019-0391-0 31700622 PMC6827230

[CIT0021] Huang, S., J. Wei, H. Yu, X. Hao, J. Zuo, C. Tan, and J. Deng. 2020. Effects of dietary fiber sources during gestation on stress status, abnormal behaviors and reproductive performance of sows. Animals. 10:131–141. doi:10.3390/ani10010141 31952304 PMC7022560

[CIT0022] Huntley, N. F., and J. F. Patience. 2018. Xylose metabolism in the pig. PLoS One 13:e0205913. doi:10.1371/journal.pone.0205913 30359396 PMC6201911

[CIT0062] Hurum, D., & Rohrer, J. (2016). Determination of carbohydrates in urine by HPAE-PAD. Sunnyvale: Thermo Fisher Scientific.

[CIT0023] Jaworski, N. W., H. N. Lærke, K. E. Back Knudsen, and H. H. Stein. 2015. Carbohydrate composition and in vitro digestibility of dry matter and non-starch polysaccharides in corn, sorghum, and wheat, and co-products from these grains. J. Anim. Sci. 93:1103–1113. doi:10.2527/jas.2014-8147 26020887

[CIT0024] Jha, R., J. M. Fouhse, U. P. Tiwari, L. Li, and B. P. Willing. 2019. Dietary fiber and intestinal health of monogastric animals. Front. Vet. Sci. 6:48. doi:10.3389/fvets.2019.00048 30886850 PMC6409295

[CIT0025] Kerr, B. J., and R. A. Easter. 1995. Effect of feeding reduced protein, amino acid-supplemented diets on nitrogen and energy balance in grower pigs. J. Anim. Sci. 73:3000–3008. doi:10.2527/1995.73103000x 8617671

[CIT0026] Kerr, B. J., and G. C. Shurson. 2013. Strategies to improve fiber utilization in swine. J. Anim. Sci. Biotechnol. 4:1–12. doi:10.1186/2049-1891-4-11 23497595 PMC3623846

[CIT0027] Knudsen, K. B. 2001. The nutritional significance of “dietary fibre” analysis. Anim. Feed Sci. Technol. 90:3–20. doi:10.1016/S0377-8401(01)00193-6

[CIT0028] Li, Q., E. R. Burrough, N. K. Gabler, C. L. Loving, O. Sahin, S. A. Gould, and J. F. Patience. 2019a. A soluble and highly fermentable dietary fiber with carbohydrases improved gut barrier integrity markers and growth performance in F18 ETEC challenged pigs. J. Anim. Sci. 97:2139–2153. doi:10.1093/jas/skz093 30888017 PMC6488326

[CIT0029] Li, Y., H. Liu, L. Zhang, Y. Yang, Y. Lin, Y. Zhuo, Z. Fang, L. Che, B. Feng, S. Xu, et al. 2019b. Maternal dietary fiber composition during gestation induces changes in offspring antioxidative capacity, inflammatory response, and gut microbiota in a sow model. Int. J. Cell Sci. Mol. Biol. 21:20–31. doi:10.3390/ijms21010031 PMC6981455 31861629

[CIT0030] Long, S., J. Hu, S. Mahfuz, H. Ma, and X. Piao. 2021. Effects of dietary supplementation of compound enzymes on performance, nutrient digestibility, serum antioxidant status, immunoglobulins, intestinal morphology and microbiota community in weaned pigs. Arch. Anim. Nutr. 75:31–47. doi:10.1080/1745039X.2020.1852008 33317350

[CIT0031] McPherson, R. L., F. Ji, G. Wu, J. R. Blanton Jr, and S. W. Kim. 2004. Growth and compositional changes of fetal tissues in pigs. J. Anim. Sci. 82:2534–2540. doi:10.2527/2004.8292534x 15446468

[CIT0032] Melo-Duràn, D., D. Solà-Oriol, S. Villagomez-Estrada, and J.F. Pérez. 2019. Enzymes as an alternative to antibiotics: an overview. In: G. Gonzalez-Ortiz, M.R. Bedford, K. E. Bach Knudsen, C. Courtin and H.L. Classen, editors, The value of fibre. Engaging the second brain for animal nutrition. Wageningen, The Netherlands: Wageningen Academic Press; p. 272–286.

[CIT0033] Meng, L., Z. Song, A. Liu, U. Dahmen, X. Yang, and H. Fang. 2021. Effects of lipopolysaccharide-binding protein (LBP) single nucleotide polymorphism (SNP) in infections, inflammatory diseases, metabolic disorders and cancers. Front. Immunol. 12:681810–681810. doi:10.3389/fimmu.2021.681810 34295331 PMC8290185

[CIT0034] Miller, N. J., C. Rice-Evans, M. J. Davies, V. Gopinathan, and A. Milner. 1993. A novel method for measuring antioxidant capacity and its application to monitoring the antioxidant status in premature neonates. Clin. Sci. 84:407–412. doi:10.1042/cs0840407 8482045

[CIT0035] Mishra, A., and G. K. Makharia. 2012. Techniques of functional and motility test: how to perform and interpret intestinal permeability. J. Neurogastroenterol. Motil. 18:443–447. doi:10.5056/jnm.2012.18.4.443 23106006 PMC3479259

[CIT0036] Moehn, S., Franco, D., Levesque, C., Samuel, R. and Ball, R.O., 2012. Phase feeding for pregnant sows. Edmonton, Alberta, Canada: Swine Research and Technology Center, p. 4–10.

[CIT0063] Navarro, D. M., E. M. Bruininx, L. de Jong, and H. H. Stein. 2018. Effects of physicochemical characteristics of feed ingredients on the apparent total tract digestibility of energy, DM, and nutrients by growing pigs. J. Anim. Sci. 96(6):2265-2277. doi:10.1093/jas/sky149. 29688508 PMC6095346

[CIT0037] Noblet, J., J. Y. Dourmad, and M. Etienne. 1990. Energy utilization in pregnant and lactating sows: modeling of energy requirements. J. Anim. Sci. 68:562–572. doi:10.2527/1990.682562x 2179194

[CIT0038] Noblet, J., H. Fortune, X. S. Shi, and S. Dubois. 1994. Prediction of net energy value of feeds for growing pigs. J. Anim. Sci. 72:344–354. doi:10.2527/1994.722344x 8157519

[CIT0039] NRC. 2012. Nutrient requirements of swine. 11th rev. ed. Washington, DC: Natl. Acad. Press.

[CIT0040] Oresanya, T. F., A. D. Beaulieu, E. Beltranena, and J. F. Patience. 2007. The effect of dietary energy concentration and total lysine/digestible energy ratio on the growth performance of weaned pigs. Can. J. Anim. Sci. 87:45–55. doi:10.4141/a05-064

[CIT0041] Oryschak, M. A., P. H. Simmins, and R. T. Zijlstra. 2002. Effect of dietary particle size and carbohydrase and/or phytase supplementation on nitrogen and phosphorus excretion of grower pigs. Can. J. Anim. Sci. 82:533–540. doi:10.4141/a02-016

[CIT0042] Petry, A. L., and J. F. Patience. 2020. Xylanase supplementation in corn-based swine diets: a review with emphasis on potential mechanisms of action. J. Anim. Sci. 98:skaa318. doi:10.1093/jas/skaa318 32970148 PMC7759750

[CIT0043] Petry, A. L., N. F. Huntley, M. R. Bedford, and J. F. Patience. 2020. Xylanase increased the energetic contribution of fiber and improved the oxidative status, gut barrier integrity, and growth performance of growing pigs fed insoluble corn-based fiber. J. Anim. Sci. 98:1–11. doi:10.1093/jas/skaa233 PMC7392531 32687554

[CIT0044] Petry, A. L., J. F. Patience, L. R. Koester, N. F. Huntley, M. R. Bedford, and S. Schmitz-Esser. 2021a. Xylanase modulates the microbiota of ileal mucosa and digesta of pigs fed corn-based arabinoxylans likely through both a stimbiotic and prebiotic mechanism. PLoS One 16:e0246144. doi:10.1371/journal.pone.0246144 33503052 PMC7840016

[CIT0045] Petry, A. L., J. F. Patience, N. F. Huntley, L. R. Koester, M. R. Bedford, and S. Schmitz-Esser. 2021b. Xylanase supplementation modulates the microbiota of the large intestine of pigs fed corn-based fiber by means of a stimbiotic mechanism of action. Front. Microbiol. 12:619970. doi:10.3389/fmicb.2021.619970 33841350 PMC8024495

[CIT0046] Renteria-Flores, J. A., L. J. Johnston, G. C. Shurson, and D. D. Gallaher. 2008. Effect of soluble and insoluble fiber on energy digestibility, nitrogen retention, and fiber digestibility of diets fed to gestating sows. J. Anim. Sci. 86:2568–2575. doi:10.2527/jas.2007-0375 18539846

[CIT0047] Samuel, R. S., S. Moehn, P. B. Pencharz, and R. O. Ball. 2012. Dietary lysine requirement of sows increases in late gestation. J. Anim. Sci. 90:4896–4904. doi:10.2527/jas.2011-4583 23048137

[CIT0048] Seethaler, B., N. K. Nguyen, M. Basrai, M. Kiechle, J. Walter, N. M. Delzenne, and S. C. Bischoff. 2022. Short-chain fatty acids are key mediators of the favorable effects of the Mediterranean diet on intestinal barrier integrity: data from the randomized controlled LIBRE trial. Am. J. Clin. Nutr. 116:928–942. doi:10.1093/ajcn/nqac175 36055959

[CIT0049] Serdar, Z., E. Gür, M. Çolakoðullarý, O. Develioðlu, and E. Sarandöl. 2003. Lipid and protein oxidation and antioxidant function in women with mild and severe preeclampsia. Arch. Gynecol. Obstet. 268:19–25. doi:10.1007/s00404-002-0302-y 12673470

[CIT0050] Shang, Q., S. Liu, H. Liu, S. Mahfuz, and X. Piao. 2021. Impact of sugar beet pulp and wheat bran on serum biochemical profile, inflammatory responses and gut microbiota in sows during late gestation and lactation. J. Anim. Sci. Biotechnol. 12:1–14. doi:10.1186/s40104-021-00573-3. 33879267 PMC8059298

[CIT0060] Tiwari, U. P., H. Chen, S.W. Kim, and R. Jha. 2018. Supplemental effect of xylanase and mannanase on nutrient digestibility and gut health of nursery pigs studied using both in vivo and in vitro models. Anim Feed Sci Technol. 245:77-90. doi:10.1016/j.anifeedsci.2018.07.002.

[CIT0052] Torres-Pitarch, A., D. Hermans, E. G. Manzanilla, J. Bindelle, N. Everaert, Y. Beckers, D. Torrallardona, G. Bruggeman, G. E. Gardiner, and P. G. Lawlor. 2017. Effect of feed enzymes on digestibility and growth in weaned pigs: a systematic review and meta-analysis. Anim. Feed Sci. Technol. 233:145–159. doi:10.1016/j.anifeedsci.2017.04.024

[CIT0053] Tsai, T., C. R. Dove, P. M. Cline, A. Owusu-Asiedu, M. C. Walsh, and M. Azain. 2017. The effect of adding xylanase or β-glucanase to diets with corn distillers dried grains with solubles (CDDGS) on growth performance and nutrient digestibility in nursery pigs. Livest. Sci. 197:46–52. doi:10.1016/j.livsci.2017.01.008

[CIT0054] Van Soest P , Robertson J. 1979. Systems of analysis for evaluating fibrous feeds. In Standardization of analytical methodology for feeds: proceedings. IDRC.

[CIT0056] Williams, B. A., L. J. Grant, M. J. Gidley, and D. Mikkelsen. 2017. Gut fermentation of dietary fibres: physico-chemistry of plant cell walls and implications for health. Int. J. Mol. Sci. 18:2203. doi:10.3390/ijms18102203 29053599 PMC5666883

[CIT0057] Zeng, Z. K., Q. Y. Li, Q. Y. Tian, Y. T. Xu, and X. S. Piao. 2018. The combination of carbohydrases and phytase to improve nutritional value and non-starch polysaccharides degradation for growing pigs fed diets with or without wheat bran. Anim. Feed Sci. Technol. 235:138–146. doi:10.1016/j.anifeedsci.2017.11.009

[CIT0058] Zervas, S., and R. T. Zijlstra. 2002. Effects of dietary protein and fermentable fiber on nitrogen excretion patterns and plasma urea in grower pigs. J. Anim. Sci. 80:3247–3256. doi:10.2527/2002.80123247x 12542166

